# Poxviral Targeting of Interferon Regulatory Factor Activation

**DOI:** 10.3390/v12101191

**Published:** 2020-10-20

**Authors:** Clara Lawler, Gareth Brady

**Affiliations:** Trinity Translational Medicine Institute, St James’ Campus, Trinity College Dublin, D08 W9RT Dublin, Ireland

**Keywords:** poxvirus, innate immune response, virus-host interaction, immune evasion, interferon regulatory factor

## Abstract

As viruses have a capacity to rapidly evolve and continually alter the coding of their protein repertoires, host cells have evolved pathways to sense viruses through the one invariable feature common to all these pathogens—their nucleic acids. These genomic and transcriptional pathogen-associated molecular patterns (PAMPs) trigger the activation of germline-encoded anti-viral pattern recognition receptors (PRRs) that can distinguish viral nucleic acids from host forms by their localization and subtle differences in their chemistry. A wide range of transmembrane and cytosolic PRRs continually probe the intracellular environment for these viral PAMPs, activating pathways leading to the activation of anti-viral gene expression. The activation of Nuclear Factor Kappa B (NFκB) and Interferon (IFN) Regulatory Factor (IRF) family transcription factors are of central importance in driving pro-inflammatory and type-I interferon (TI-IFN) gene expression required to effectively restrict spread and trigger adaptive responses leading to clearance. Poxviruses evolve complex arrays of inhibitors which target these pathways at a variety of levels. This review will focus on how poxviruses target and inhibit PRR pathways leading to the activation of IRF family transcription factors.

## 1. Introduction

Germline-encoded pattern recognition receptors (PRRs) ‘sense’ infection by binding invariable chemical features of invading pathogens called pathogen-associated molecular patterns (PAMPs). Each PRR activates signal transduction pathways leading to gene expression, which orchestrates clearance of the specific type of pathogen from which the PAMP was derived. The principle PAMPs of viruses are their nucleic acids, which are distinguished from native host nucleic acids by cellular location and subtle differences in chemistry [1]. In order to evade or suppress anti-viral immunity, viruses invariably evolve targeted strategies to prevent activation of these sensing systems at a variety of points along the pathway to sensing-induced gene expression. Anti-viral PRRs typically drive expression of type I interferons (TI-IFNs) and pro-inflammatory cytokines, such as interleukin−1 (IL−1) and tumor necrosis factor (TNF), which heavily rely on the activity of IFN Regulatory Factor (IRF) and Nuclear Factor Kappa B (NFκB) family transcription factors. The induction of TI-IFNs requires the combined activity of NFκB and IRFs [2,3], whilst the regulation of pro-inflammatory genes has a stronger reliance on NFκB activity [4].

Poxviruses comprise a diverse family of large, enveloped, double-stranded DNA viruses. Their genomes range between 134 and 365 kb, which are spatially organized into central, relatively conserved regions which typically encode everything required for basic life cycle functions and diverse terminal regions with a higher degree of genetic plasticity where immunomodulatory proteins tend to be encoded. Approximately 130 to 328 open reading frames (ORFs) are bidirectionally encoded throughout their genomes [5,6]. Poxviruses broadly group into two subfamilies: Chordopoxvirinae, which infect vertebrates, and Entomopoxvirinae, which infect invertebrates. Chordopoxvirinae subdivide into ten genera: orthopoxviruses, leporipoxviruses, yatapoxviruses, parapoxviruses, cervidpoxviruses, capripoxviruses, suipoxviruses, molluscipoxviruses, crocodylipoxviruses and avipoxviruses. They can be also grouped into four phylogenetic categories by order of divergence [7]. Group I is the most divergent and includes the Avipoxvirus genera with Fowlpox (FPV) and Canarypox viruses. Group II, the next most divergent, includes Molluscipoxvirus with Molluscum Contagiosum virus (MCV) and Parapoxvirus (PPV) genera. The remaining two groups, III and IV, are closely clustered together, often being referred to as ‘sister groups’ based on the relative proximity of their phylogenetic grouping. Group III comprises members of Capripoxvirus, Leporipoxvirus, such as myxoma virus (MYXV), Suipoxvirus and Yatapoxvirus genera and Group IV includes the seven members of the Orthopoxvirus genera, such as camelpoxvirus (CMPV), variola virus (VARV), vaccinia virus (VACV), monkeypox virus (MPV), ectromelia virus (ECTV) and cowpox virus (CPV) which are arguably the best characterized of the poxviruses.

Poxviruses have well-characterized immunoevasive and immunomodulatory strategies to suppress activation of the host innate immune system, seeking to sense them in order to drive effector responses leading to their clearance [8]. These strategies target signaling pathways at a variety of points in activation, with a preference for targeting downstream at common points of convergence in the activation of NFκB and IRF family transcription factors. These transcription factors collaborate in transactivating a wide range of target genes, but the NFκB family bias towards pro-inflammatory gene regulation and the IRF family towards interferon gene induction. By inhibiting activation of both these transcription factors with both discrete and multi-functional inhibitors, poxviruses can delay or silence pro-inflammatory and interference responses, depending on how efficiently targeting evolves to be.

Poxviruses appear to have a larger number of dedicated inhibitors focused on the inhibition of NFκB-activating pathways [8] but less dedicated IRF-targeting inhibitors have been discovered thus far. This could suggest that (a) IRF signaling is easier to inhibit with less targeting inhibitors, (b) the requirement for NFκB in TI-IFN regulation makes dedicating inhibitors to IRFs less important or (c) that inhibiting inflammation is a higher priority for these viruses. Poxviral inhibitors of NFκB activation have been covered at length in other reviews [8,9]. In this review, we will discuss the strategies that poxviruses have evolved to target pathways leading to the activation of IRF-family transcription factors, with a focus on preventing the induction of TI-IFNs.

## 2. Poxviral Targeting of the IRF Family and Their Activation Complexes

The IRF family of transcription factors contains nine members in most vertebrates (IRF-1 to -9) [10,11,12]. An additional family member, IRF-10, was identified in chickens but is absent in humans and mice [13]. All IRF family members share a highly conserved amino-terminal DNA-binding domain (DBD) (of approximately 120 amino acids) which possesses a helix-loop-helix structure and a motif containing five tryptophan residues, similar to that of Myb family transcription factors which bind IFN-stimulated response elements (ISREs) with the consensus sequence A/GNGAAANNGAAACT [14]. Conversely, the carboxy-terminal is more diverse and contains IRF association domains (IAD) 1 and 2, which facilitate specific homo- and heterodimeric associations between family members, other transcription factors and co-factors required for transactivation to form activating or repressive complexes [10,11,12].

Although other IRF family members were initially thought to be responsible for TI-IFN induction, IRF3 and IRF7 are now considered to be the principle family members involved downstream of cytosolic nucleic acid receptors and nucleic acid sensing Toll-like receptors (TLRs) (TLR3, -4, -7, -8 and -9) [15]. Whilst IRF3 shows a relatively ubiquitous pattern of expression, IRF7 expression is typically weak in unstimulated cells, with the exception of plasmacytoid dendritic cells (pDCs) but can be strongly induced downstream of IRF3-induced TI-IFN stimulation, which bolsters further IFN induction [3,16]. Thus, IRF3 plays a stronger role in primary sensing of viral PAMPs in most cells for early sensing of viruses.

**Figure 1:**
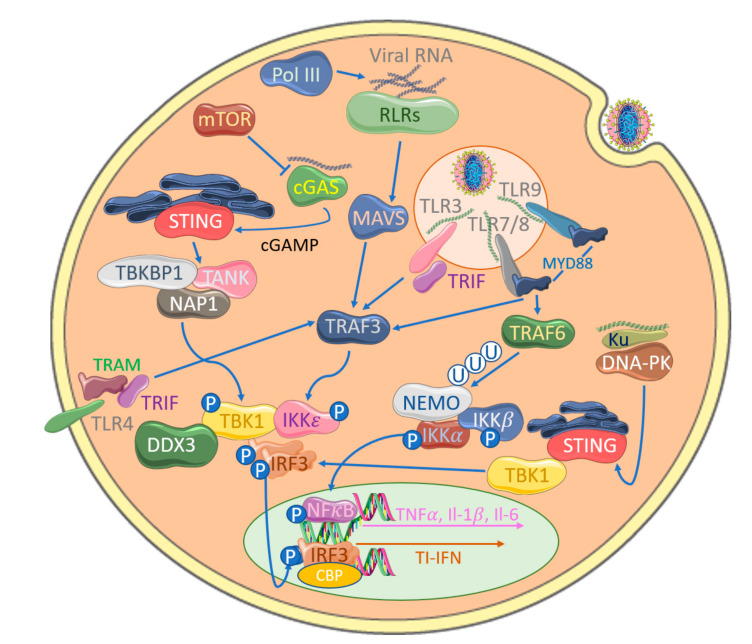
Detection of poxviruses by anti-viral pattern recognition receptors (PRRs) leads to the activation of IRFs. IRF3 phosphorylation by TBK1 and IKKε leads to its dimerization and nuclear translocation, where it associates with CBP/p300 to initiate transcription of anti-viral target genes, such as type I interferons (TI-IFNs). TRAF3 plays a central role in Toll-like receptor (TLR) signaling through generation of activating polyubiquitin chains. TLR3 sensing of dsRNA recruits TRIF, which activates TRAF3, in turn forming a complex with TBK1 and IKKε to promote IRF3 phosphorylation. The MyD88 adaptor protein associates with TLR7/8 and TLR9 is also able to activate TRAF3-mediated signaling. TLR4-associated TRAM can additionally activate this pathway through associations with TRIF. NFκB essential modulator (NEMO) is traditionally associated with NFκB activation to induce pro-inflammatory cytokine production but can also activate TBK1, thus inducing IRF3 phosphorylation. RIG-I-like receptors (RLRs) sense viral RNA and activate a common activator mitochondrial antiviral signaling protein (MAVS), leading to IRF activation. Cytosolic DNA sensing by cGAS, the RLR RIG-I (potentially via DNA Pol III-generated transcript from viral DNA) or Ku additionally activates this system, leading to IRF3 phosphorylation.

IRF3 is activated downstream of all anti-viral PRRs through phosphorylation by IκB kinase (IKK)-related protein kinases, TANK-binding Kinase (TBK1) and IKKε, resulting in its dimerization and translocation into the nucleus in association with CREB-binding protein (CBP)/p300 to bind and transactivate the expression of target genes, such as TI-IFNs [17,18,19,20] (Figure 1). A model for IRF3 activation has been suggested based on existing structural and biochemical evidence, whereby activation is regulated by multiple phosphorylation events within its eight C-terminal Ser/Thr residues, ^385^SSLENTVDLHISNSHPLSLTS^405^. This region is functionally separated into two sites: site I includes Ser^385^ and Ser^386^, whereas site II includes Ser^396^, Ser^398^, Ser^402^, Thr^404^ and Ser^405^. Although the precise residues modified in vivo remain controversial, initial phosphorylation in site II on Thr^404^ or Ser^405^ by TBK1 relieves a state of auto-inhibition and allows association with CBP/p300. In this quasi-active state, IRF3 is then primed to be fully activated by a second phosphorylation by TBK1 on Ser^385^ and Ser ^386^ within site I and then translocates into the nucleus [21,22,23,24].

While the IKK complex that regulates canonical NFκB activation has been well-characterized, the precise nature of the equivalent IRF-activating complexes remains unclear. NFκB activation is controlled by an IKK complex which consists of a regulatory protein NFκB essential modulator (NEMO) and two IKK-family subunits, IKKα and IKKβ. Activation of this large multimeric complex begins with the binding of a ubiquitin-binding domain within NEMO to di-ubiquitin from TRAF6-generated ubiquitin chains, both free and conjugated, to multiple proteins in the signaling pathway. Biochemical and structural data suggest that this binding induces a conformational change within NEMO, which primes IKKβ for activation by allowing initial trans-auto phosphorylation, which in turn allows subsequent phosphorylation by transforming growth factor b-activated kinase−1 (TAK1), resulting in a fully active state (reviewed in [25]).

TBK1 was discovered based on its interaction with TANK [26] and was initially described as an NFκB-activating kinase. This was due to the fact that it could phosphorylate IKKβ in vitro and *tbk1*-deficient mice phenotypically resembled p65-, IKKβ- and NEMO-deficient mice with profound liver apoptosis in utero [27,28,29,30,31]. IKKε was identified as an inducible kinase (IKKi) related to IKKα and IKKβ [32]. Subsequent studies clarified the roles of both TBK1 and IKKε as key kinases in the stimulus-inducible phosphorylation of IRF3 and IRF7 in both viral and bacterial infection [17,18,33]. Single and double TBK1/IKKε knockout mice and chemical inhibition have demonstrated that although TBK1 and, to a much lesser extent, IKKε have an essential role in IRF3-mediated IFNβ production, they are not required for NFκB activation in response to Toll-like receptors (TLRs), IL−1β or TNFα [33,34,35]. However, there is evidence that they do play a role, albeit redundantly, in NFκB activation in DNA sensing through the stimulator of interferon genes (STING) pathway [36].

Multiple TNF Receptor Associated Factors (TRAFs) have been shown to be involved in IRF-activating pathways (TRAF2, -3, -5 and -6) [37], although TRAF3 appears to more commonly play this role in most cells in vivo with TLR-dependent and -independent signaling to IRFs [38,39,40,41]. TRAF3 appears to play a similar role to TRAF6 in IKK complex activation by generating activating ubiquitin chains [42,43]. Although NEMO has been shown to play a role in TI-IFN induction, not solely for the NFκB contribution to IFNβ transactivation but also for activation of TBK1 and phosphorylation of IRF3 [17,19], other adapters have been suggested to play NEMO-like roles in IRF-activating complexes, such as Optineurin (OPTN) (TBK1 only) [44], TANK [26], NFκB-activating protein (NAP1) [45] and TBK1 Binding Protein 1 (TBKBP1) [46]. Unlike in the model of IKK complex activation of NFκB, NEMO does not directly associate with TBK1 and IKKε, whereas the other adapters bind these IRF-activating kinases in a mutually exclusive fashion, suggesting multiple discrete complexes are involved. However, TANK has also been shown to bridge an association of NEMO to TBK1 and IKKε in some contexts and cell types [47,48].

The nature of the complex(es) formed for the activation of IRFs may be highly cell type-dependent. For example, recent evidence suggests that NAP1, TANK and TBKBP1 are not required for the RIG-I-like receptor (RLR) mitochondrial antiviral signaling (MAVS) pathway in HEK293T cells [37]. Additionally, although NEMO-TANK-TBK1/IKKε complexes form in macrophages, virus-induced IFNβ is not impaired in TANK-deficient cells [49,50]. Thus, the available evidence to date suggests that IRF-activating complexes may be significantly more diverse and context-dependent than the system that activates canonical NFκB, and much work remains to be done in clarifying the nature of these complexes, their regulation and their relative importance by cell type and stimulus. How this reflects the relative abundance of dedicated poxviral NFκB pathways inhibitors and the scarcity of those targeting IRF activation also remains unclear.

**Figure 2:**
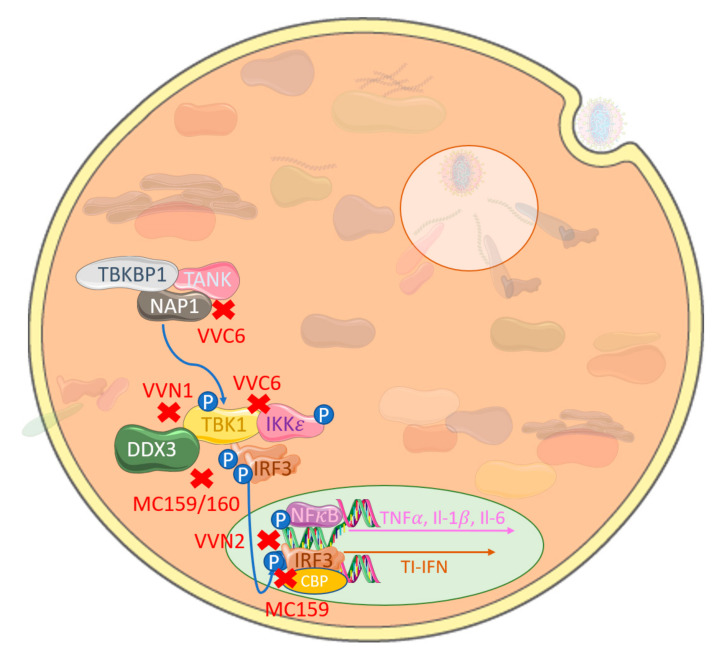
Poxviruses commonly inhibit interferon regulatory factor (IRF) signaling by targeting proximal activation complexes or IRFs directly. VACV-encoded C6 (VVC6) associates with multiple IRF3-activating adaptor proteins and kinase; TBKBP1, TANK, NAP1, TBK1 and IKKe specifically inhibit IFNβ production independent of NFκB activation. VACV-encoded N1 (VVN1) inhibits TBK1 activation, with a parallel function seen in MCV-encoded MC159 and MC160. VACV-encoded N2 (VVN2) associates with phosphorylated IRF3 in the nucleus to inhibit initiation of gene transcription. Similarly, MCV-encoded MC159 blocks nuclear IRF3 association with CBP/p300.

Nevertheless, analogous to how poxviruses have invested considerable evolution to targeting the IKK complex and NFκB directly [8], inhibition of IRF activation is commonly directed at these same downstream points in order to broadly inhibit activation of this family of IFN-driving transcription factors (Figure 2). VACV N1 was initially shown to inhibit TLR-driven NFκB signaling by targeting the IKK complex; this inhibitor also inhibits IRF3 signaling by targeting TBK1 [51,52]. VACV C6 protein has been shown to inhibit IRF3 activation by interacting with both the adaptors NAP1, TANK and TBKBP1 as well as TBK1 and IKKε kinases [53]. As these activation complexes are a central point of convergence for all upstream sensing pathways that activate IRFs, it is unsurprising that C6 was shown to inhibit RNA, DNA and virus-induced IFNβ production, as well as IRF3/7 activation, without affecting activation of NFκB or its target genes. Interestingly, C6 has also been shown to target further downstream in the viral interference system by inhibiting IFN-signaling itself by binding to the transactivation domain of STAT2, preventing its ability to transactivate at IFN-inducible promoters [54], thus multifunctionally targeting nuclear components as well as cytosolic TI-IFN-inducing components. C6 additionally targets transcriptional regulation by degrading histone deacetylase 5 (HDAC5) through E3 ubiquitin ligase [55].

MCV is the only extant poxvirus that appears to have specifically adapted to infect humans [56,57]. MCV infections are common throughout the developed world and it is the primary poxvirus causing human disease currently in circulation [57]. One striking feature of this virus is the mild and mostly uninflamed nature of its virus-filled lesions in the skin over long periods of infection, suggesting that this virus has evolved very efficient inhibitors of human innate pathways that may offer insights into how to target them in diseases driven by inflammation. A relatively small number of MCV inhibitors have been described to date, some of which target IRF activation. The MCV viral FLICE Inhibitory Proteins (FLIPs) MC159 and MC160 have been previously shown to inhibit both NFκB activation and apoptosis [58,59,60]. Both were subsequently shown to prevent TBK1/IKKε activation and phosphorylation of both TBK1 and IRF7. Interestingly, these mechanisms were independent of their other inhibitory activities and only MC159 directly associated with TBK1 [61] (Figure 2). In addition to this, MC159 was also shown to prevent the interaction of IRF3-CBP/p300 with target promoters [62]. Given the density of inhibitors in other well-characterized poxviruses, it is likely that additional IRF inhibitors remain to be discovered in this highly understudied human-adapted poxvirus.

The VACV bcl−2-family protein N2 inhibits IRF activity directly at the level of nuclear IRF3 [63] (Figure 2). These authors observed that N2 had no effect on upstream signaling and did not inhibit NFκB pathway activation. They also observed that it did not affect nuclear translocation of IRF3 and that it co-localized with IRF3 in the nucleus. As N2 inhibited virus-induced CXCL10, which has an IRF3-dependant promoter, this suggests that N2 prevents IRF3-dependant gene regulation, although the precise mechanism of inhibition has not yet been described. Similar functions are seen in measles virus C protein [64] and Nipah virus W protein [65].

## 3. Poxviral Targeting of TLR-Induced IRF Activation

**Figure 3:**
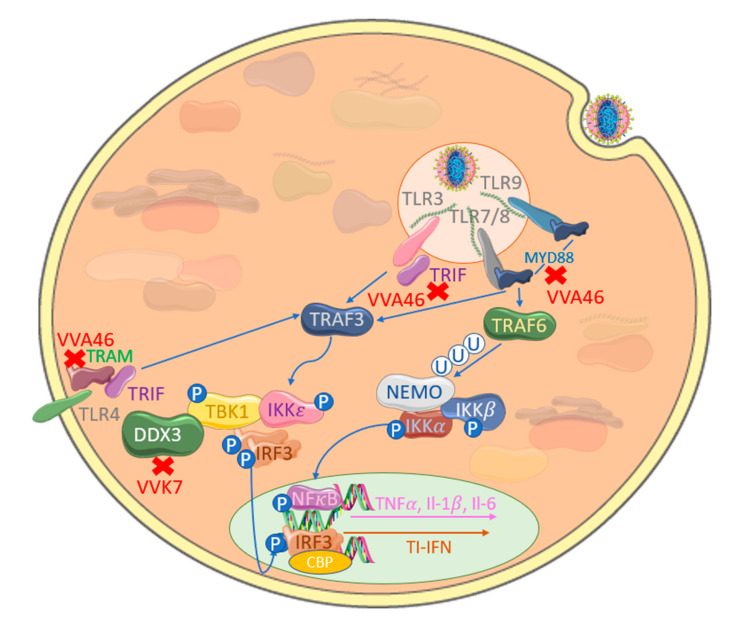
TLR adaptor-targeting by poxviruses inhibits TLR-mediated IRF activation. VACV-encoded A46 (VVA46) interacts with the Toll Il−1R (TIR) domain of MyD88, TRIF and TRAM, inhibiting IRF activation. Additionally, VACV-encoded K7 (VVK7) binds the N-terminus of DDX3, blocking TBK1 and IRF3 activation.

TLRs are the prototypical PRRs for PAMPs and damage-associated molecular patterns (DAMPs) and are widely conserved in animals [66]. TLRs sense the presence of poxviruses when cells engage with extracellular virus particles within the endosomes of phagocytotic macrophages and dendritic cells at infection sites or after endocytosis and uncoating of virus during infection of multiple cell types (Figure 3).

TLR3 recognizes viral double-stranded RNA (dsRNA) in endosomes and triggers signaling through homodimeric interactions of the TLR3 Toll-IL1 Receptor (TIR) domain and the adapter TIR-domain-containing adapter-inducing IFNβ (TRIF), leading to recruitment of TRAF3 which undergoes K63-linked self-ubiquitination, resulting in complex formation with TBK1 and IKKε, which in turn phosphorylate and activate IRF3 [67]. Involvement of TLR3 in responding to VACV infection has been demonstrated and appears to drive aspects of immunopathology of this infection in mouse models, even though lower levels of virus replication are observed in TLR3^−/-^ mice. This highlights the critical balance between preventative and over-zealous innate anti-viral responses [68]. As the opposite effect was seen in TRIF^−/-^ mice, this suggests a TRIF-dependent TLR other than TLR3 may paradoxically be protective in VACV infections [69]. Consistent with this, TLR4 can also use TRIF for signaling. Although best known as the signaling receptor for LPS from gram negative bacteria, TLR4 was shown to play a protective role during VACV pulmonary infection, the mechanism for this remains unclear [69]. As TLR4 was shown to sense glycoproteins from respiratory syncytial virus (RSV) [70] and vesicular stomatitis virus (VSV) [71], this suggests that it may also sense VACV surface glycoproteins in a similar manner. An alternative explanation is that TLR4 is simply sensing DAMPs generated during infection-associated tissue damage, as has been observed in influenza virus infection [72]. There have been suggestions that TLR2 also plays a role in sensing VACV and Ectromelia infection [73]; however, other authors have not observed this with VACV infection in mouse infection models [74].

TLR9, the first DNA-sensing PRR to be discovered [75], senses unmethylated CpG DNA, whilst TLR7 and TLR8 sense single-stranded RNA [66]. These TLRs activate IRF7 via Myeloid differentiation primary response gene 88 (MyD88), TRAF6 and TRAF3 [66]. Poxvirus infection is detected by TLR7 [76], TLR8 [77] and TLR9 [78,79] in endosomes of pDCs and conventional dendritic cells (cDCs). Survival of mice after a lethal ECTV infection was critically dependent on TLR9, while MVA infection protected mice from ECTV in a manner also dependent on TLR9 [78]. Recognition of poxvirus by TLR7 was proposed to involve detection of viral RNA transcripts [76], while, surprisingly, TLR8-dependent responses were suggested to be mediated by recognition of poly(A)/(T)-rich poxviral DNA sequences [77]. Although TLR3 and TLR9 are not normally expressed in skin [80,81], MCV-infected skin lesions have increased expression of TLR3 and TLR9 in adjacent-to-skin papules, suggesting that these TLRs may be involved in the local host response to MCV in infected keratinocytes [81,82].

Consistent with the role of TLRs in sensing poxviruses, VACV encodes proteins that inhibit TLR-driven IRF activation (Figure 3). The VACV TLR inhibitor A46 was identified as having sequence similarity to the TIR domain used by TLRs to signal to their adaptors [83] but was subsequently shown to be a member of the wider poxviral bcl−2 family, which was confirmed by determination of its crystal structure [84,85]. A46 inhibits TLR4-, TLR3- and TRIF-activated IRF3/7 [83,86]. It inhibits by directly targeting the complexes assembled on TLRs by interacting with the TIR domain in both TLRs and their adaptor proteins MyD88, TRIF, MyD88-adapter-like (Mal) and TRIF-related adapter molecule (TRAM) and prevents adaptor recruitment to the TLR4 complex [86,87]. Interestingly, inhibition of TLR4 could be mimicked using a cell-penetrating peptide (viral inhibitor peptide of TLR4, or VIPER) based on the surface of A46 that is required to disrupt TLR2- and -4-signaling through TRAM to activate IRF3 [88,89,90]. Another VACV bcl−2 protein, K7, inhibits both TLR3- and -4-stimulated IRF3 phosphorylation as well as IRF3 and-7 activation [91] by binding to the N-terminus of DDX3 helicase in both cytoplasmic and nuclear compartments [92]. Interestingly, DDX3 targeting is also convergently employed by the hepatitis B virus encoded polymerase [93] and the hepatitis C virus core protein [94] as well as the well characterized HIV rev protein [95].

Other poxviral proteins have unexpected roles in inhibiting TLR signaling to IRF activation. For example, E3 is a key virulence factor for VACV that has multiple activities in suppressing the host immune response and contains an N-terminal Z-DNA/RNA-binding motif as well as a C-terminal dsRNA-binding domain [96]. The Z-DNA/RNA-binding motif of E3 was shown to block TLR7-driven induction of IFNα via IRF7 and the activation of pDCs [76]. This domain is not retained in the Myxoma orthologue of E3, which prevents it from evading TLR7, highlighting a common phenomenon of gain or loss of function between orthologous proteins in different poxviruses.

## 4. Inhibition of IRF Activation by Cytosolic Nucleic Acid Sensors by Poxviruses

**Figure 4:**
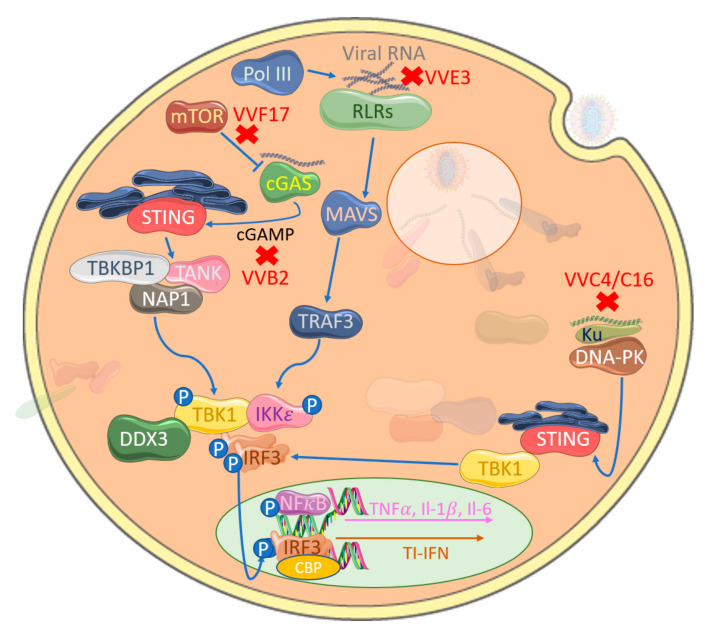
Poxviral inhibition of cytosolic nucleic acid sensing leading to IRF activation. VACV-encoded E3 (VVE3) binds dsDNA acting as a competitive inhibitor of RLR activation. Similarly, VACV-encoded C4 and C16 (VVC4/C16) inhibit DNA binding to Ku, therefore blocking DNA-PK-mediated stimulator of interferon genes (STING) activation and, hence, TBK1 activation. The VACV-encoded poxin B2 (VVB2) hydrolyses the 3′−5′ bond on cGAMP, thus inactivating this key messenger molecule in cGAS-STING activation. A further target is mTOR-dependent cGAS degradation by VACV-encoded F17 (VVF17), thus suppressing cGAS-mediated TI-IFN gene expression.

Despite the ability of TLRs to sense poxvirus infection, the attenuated modified vaccinia Ankara (MVA), which possesses several of the previously described inhibitors, still induces TI-IFNs in a TLR-independent fashion [97]. Consistent with this, a range of additional cytosolic PRRs can detect both poxviral RNA and DNA. The cytosolic RNA receptors melanoma differentiation factor 5 (MDA5) and retinoic acid-inducible gene (RIG-I) detect long dsRNA and dsRNA with a 5′ triphosphate group, respectively, in the cytoplasm of cells infected with RNA viruses [98]. Upon activation, these RLRs engage the adaptor protein MAVS, resulting in TBK1-induced IRF3 phosphorylation through TBK1 and IKKε [37] (Figure 4). Several reports demonstrate how poxviruses are also sensed by RLRs. For example, MYXV stimulates IRF-dependent TI-IFN production in primary human macrophages through RIG-I [99], while VACV induces TI-IFN in a RIG-I- and MDA5-dependent manner in different cell types and MVA-induced IFNβ and IFN-dependent chemokines via MDA5 and MAVS but not RIG-I in macrophages, suggesting both virus and cell type differences in these responses [100,101,102]. A third RLR, LGP−2, has also been shown to be important for the IRF3 activation and upregulation of IRF3-dependant genes in response to VACV DNA [103]. MVA infection also causes increased cellular expression of the RLRs, thus increasing the sensitivity of DCs to aberrant RNA [104].

The importance of RLRs in managing poxviral infection is reflected in the fact that there is evidence that poxviral infection has played a role in positive selection of RLR families in different mammalian species over time [105]. A rationale for how cytosolic dsRNA PRRs are involved in detecting poxviruses is provided by the fact that poxviruses generate large quantities of dsRNA during an infection. Although poxviral genomes are organized to cluster ORFs that express in the same direction, simultaneous transcription of both strands to generate complementary dsRNA can still occur [106]. To counter this, VACV E3, which binds dsRNA, was shown to block RLR-driven IRF3 activation in keratinocytes with E3-deleted virus, displaying increased levels of IRF3 phosphorylation [107] (Figure 4). The requirement for RLRs in anti-viral responses to poxviruses also involves the RNA polymerase III intermediate system of cytosolic DNA detection, whereby RNA polymerase III transcribes short RNA sequences from cytosolic AT-rich DNA that are direct ligands for RIG-I activation [108,109]. Interestingly, E3 can also antagonize this system [110].

Although the physiological relevance of AT-rich dsDNA-sensing by RNA polymerase III in poxviral infections is unclear, additional cytosolic DNA sensors play a central role in the potency of cytosolic DNA, whether from viral infection or from aberrant host DNA localization, to drive IRF activation and induce TI-IFNs [111]. Such DNA sensors, in many cases, strongly activate IRF3 via a well-defined STING-TBK1-IRF3 signaling axis (Figure 1), whereas the DNA-sensing cytosolic pathways to NFkB activation are still less clear. Both genetic and biochemical studies have demonstrated the importance of STING in signaling a response to DNA viruses in the cytoplasm, though how STING itself is activated by upstream DNA sensors was initially unclear [112]. A series of elegant studies then showed that cyclic-GMP-AMP (cGAMP) synthase (cGAS) is a DNA sensor upstream of STING, whose enzyme activity is stimulated by direct binding of DNA, leading to production of the novel second messenger cGAMP (reviewed in [113]). cGAMP is a direct ligand for STING, which is initially localized in the endoplasmic reticulum, but on binding, it translocates to TBK1-containing membrane-bound compartments, leading to IRF3 activation. Interestingly, after infection of cells with MVA, cGAMP can diffuse through cellular gap junctions to activate the TI-IFN response in adjacent, uninfected cells, implying that the cGAS-STING system may directly stimulate bystander cells for resistance to incoming poxviral infection [114,115]. The cGAS-STING system was also shown to sense MVA DNA in the cytoplasm of conventional DCs during infection [116]. A number of papers have demonstrated that TI-IFN induction by VACV in some cell types requires cGAS [115,116].

We have recently reviewed DNA virus inhibitors of the cGAS-STING pathway, including those of poxviruses [117]. In addition to the poxviral inhibitors that target at the level of the IRF activation or IRF activity, which inhibit this system by default, a recently discovered family of poxvirus immune nucleases (poxins) were discovered in a screen for cGAS inhibitors. The authors described how VACV B2 protein degrades cGAMP by hydrolyzing the canonical 3′–5′ bond (Figure 4) and significantly reducing IFNβ production [118]. Additionally, a component of poxviral lateral bodies expressed late in infection, F17, specifically modulates the cGAS-STING pathway to interfere with IRF-induced TI-IFN production. Targeting mTOR-dependent cGAS degradation [119] by this conserved poxvirus gene highlights precise targeting of a key viral cytosolic sensing modulatory pathway. Of interest, its late gene expression has a further target in facilitating viral protein synthesis through mTOR dysregulation [120].

Cytosolic DNA-sensing pathways outside of cGAS-STING signaling are poorly defined, but multi-layered immune defense mechanisms for every PAMP are common. A cytosolic DNA-sensing mechanism in fibroblasts has been shown to be targeted by poxviruses for immune evasion; Ferguson et al. [121] showed that DNA-dependent protein kinase (DNA-PK) senses MVA, leading to STING-dependent IRF3 activation, and DNA-PK associates with a heterodimer of Ku70 and Ku80 and is a serine/threonine protein kinase. Primarily associated with DNA damage repair, specifically double-stranded breaks, it is an emergent potential therapeutic target to enhance the success of cancer treatment [122]. However, it has also surfaced as a key component in initializing the innate immune response to viral DNA. Recently identified in humans, DNA-PK binds cytosolic DNA and acts in a STING-independent manner [123]. This pathway is antagonized by VACV through two distinct proteins, highlighting complementary multi-layered viral immune evasion mechanisms. VACV-encoded C16 is able to bind to the Ku heterodimer through its C-terminal region to block DNA binding [124] (Figure 4). A second VACV Ku-binding protein with sequence homology, C4, additionally stops DNA binding, quelling cytokine release [125]. The presence of multiple proteins targeting the same pathways directs our understanding further into these complex interactions.

Given the importance of these cytosolic sensing systems pathways for detecting poxviruses to drive TI-IFNs, as their pathways are further elaborated, along with new components and mechanisms that regulate them, we expect that additional as-yet undiscovered poxviral inhibitors that target them will also be identified.

## 5. Concluding Remarks

The induction of TI-IFNs by nucleic acid sensors is a critical feature of the response to poxviruses and indeed all viruses with differences in pathways employed, depending on the nature of the nucleic acids presented to the innate immune system during infection. These pathways have a rate-limiting reliance on IRF-family activation. Once secreted, TI-IFNs then drive IFN-stimulated gene expression in surrounding cells to induce the interference state, making uninfected cells non-permissive for the incoming virus. This IFN system aims to quarantine the virus and limit replication whilst assisting the emergence of a robust adaptive response needed for clearance. Consequently, poxviruses evolve highly efficient and, in some cases, multifunctional inhibitors which target IRF-activating pathways at multiple levels in order to prevent TI-IFN production, which would limit its spread. The extent to which they achieve this underlies the delicate balance between persistence, invasiveness and pathology that defines their presentation in disease.

## Abbreviations

PAMP	Pathogen-Associated Molecular Patterns
PRR	Pattern Recognition Receptors
NFκB	Nuclear Factor Kappa B
IFN	Interferon
IRF	Interferon Regulatory Factor
TI-IFN	Type 1 Interferon
Il−1	Interleukin 1
TNF	Tumor Necrosis Factor
FPV	Fowlpox
MCV	Molluscum Contagiosum Virus
PPV	Parapoxvirus
MYXV	Myxoma Virus
CMPV	Camelpoxvirus
VARV	Variola Virus
VACV	Vaccinia Virus
MPV	Monkey Pox Virus
ECTV	Ectromelia Virus
CPV	Cowpox Virus
CPV	Cowpox Virus
TLR	Toll-Like Receptors
IKK	I KappaB Kinase
TBK1	TANK binding kinase 1
CBP	CREB Binding Protein
NEMO	Nuclear Factor Kappa B essential modulator
TRAF	TNF Receptor Associated Factors
NAP1	Nuclear Factor Kappa B Activating Protein
FLIPS	viral FLICE Inhibitory Proteins
STING	stimulator of interferon genes
TIR	Toll Il−1R
TRIF	TIR-domain-containing adapter-inducing interferon-β
MyD88	Myeloid differentiation primary response gene 88
MAL	MyD88-adapter-like
TRAM	TRIF-related adapter molecule
MDA5	melanoma differentiation factor 5
RIG−1	retinoic acid-inducible gene
RLR	retinoic acid-inducible gene-I-like receptors
MAVS	Mitochondrial Antiviral Signaling Protein
GAS	cyclic GMP AMP Synthase
GAMP	cyclic GMP-AMP
DNA-PK	DNA-dependent protein kinase
